# The Cross-Talk between Polyphenols and the Target Enzymes Related to Oxidative Stress-Induced Thyroid Cancer

**DOI:** 10.1155/2022/2724324

**Published:** 2022-05-05

**Authors:** Shabnam Heydarzadeh, Sima Kheradmand Kia, Maryam Zarkesh, Safura Pakizehkar, Samaneh Hosseinzadeh, Mehdi Hedayati

**Affiliations:** ^1^Department of Biochemistry, School of Biological Sciences, Falavarjan Branch Islamic Azad University, Isfahan, Iran; ^2^Cellular and Molecular Endocrine Research Center, Research Institute for Endocrine Sciences, Shahid Beheshti University of Medical Sciences, Tehran, Iran; ^3^Laboratory for Red Blood Cell Diagnostics, Sanquin, Amsterdam, Netherlands

## Abstract

The most serious hallmark step of carcinogenesis is oxidative stress, which induces cell DNA damage. Although in normal conditions ROS are important second messengers, in pathological conditions such as cancer, due to imbalanced redox enzyme expression, oxidative stress can occur. Recent studies with firmly established evidence suggest an interdependence between oxidative stress and thyroid cancer based on thyroid hormone synthesis. Indeed, a reduced antioxidant defense system might play a part in several steps of progression in thyroid cancer. Based on studies that have been conducted previously, future drug designs for targeting enzymatic ROS sources, as a single agent or in combination, have to be tested. Polyphenols represent the potential for modulating biological events in thyroid cancer, including antioxidative activity. Targeting enzymatic ROS sources, without affecting the physiological redox state, might be an important purpose. As regards the underlying chemopreventive mechanisms of natural compounds that have been discussed in other cancer models, the confirmation of the influence of polyphenols on thyroid cancer is inconclusive and rarely available. Therefore, there is a need for further scientific investigations into the features of the antioxidative effects of polyphenols on thyroid cancer. The current review illustrates the association between some polyphenols and the key enzymes that take place in oxidation reactions in developing thyroid cancer cells. This review gives the main points of the enzymatic ROS sources act and redox signaling in normal physiological or pathological contexts and supplies a survey of the currently available modulators of TPO, LOX, NOX, DUOX, Nrf2, and LPO derived from polyphenols.

## 1. Introduction

The utmost common sort of endocrine malignancies is thyroid cancer which has a rapidly increasing prevalence in many countries. According to the WHO, thyroid carcinoma is categorized into subtypes based on its histopathological characteristics. The well-differentiated thyroid carcinomas are the widespread follicular cell-derived thyroid malignancies, while poorly differentiated forms are the rare ones. Despite the appropriate prognosis, 15–20% of well-differentiated cases and mostly anaplastic types stay resistant to standard therapies, including radioactive iodine, although correction of iodine deficiency can led to the less aggressive thyroid cancer subtypes. Recently, studies have focused on the relationship between thyroid cancer and nutritional factors [1, 2]. However, mutations in the signaling pathways involved in chemotherapy-induced ROS-induced tumor resistance result in decreased treatment effectiveness or tumor recurrence following an initial antitumor response [3, 4]. As a result, the majority of chemotherapeutic agents are not recommended as a single therapy choice for advanced-stage or metastatic malignancies. While these adverse events continue to be a source of contention in cancer therapy, various innovative therapeutic strategies are being examined. Polyphenols have been studied in preclinical experimental cases to better understand their synergic interaction with therapeutic factors. It should be point out that studying the relationship between oxidative stress and cancer may suggest innovative prevention strategies and could be an innovative research strategy for thyroid cancer pathogenesis and therapy. Despite the fact that herbal compounds have been shown to have chemopreventive effects in other cancer models, this study will discuss the modulation of enzymatic ROS sources by polyphenols in thyroid cancer.

The quest for new molecular targets and treatments continues to be a significant obstacle. By targeting recognized oncogene-driven changes in signal transduction pathways implicated in the generation and progression of thyroid tumors, promising outcomes have been obtained [5]. As a result, innovative targeted medications are now being evaluated in clinical trials [6]. Other molecular abnormalities found in thyroid carcinoma cells suggest additional possible treatment targets. Recent years have seen a surge of interest in the role of reactive oxygen species in neoplastic transformation and progression. Recent studies with firmly established evidence suggest interdependence between oxidative stress and thyroid cancer indicating unregulated oxidative or antioxidative molecules, following the requirement of H_2_O_2_ generation for initiation of oxidation reactions based on thyroid hormone synthesis. When compelling evidence suggests a close correlation between oxidative stress and thyroid cancer [7], it bolsters the case for testing antioxidant compounds as innovative targeted therapies. Findings informed that a reduced antioxidant defense system may influence on the progression of thyroid cancer. Thyroid cancer tissues have been found to have a greater deficiency of antioxidant scavenging enzymes than normal thyroid tissues. Thereupon, the cancer tissue is more attackable to toxic free radicals [8]. Many investigations have evaluated the ROS scavenging activity of polyphenols which prohibited cell proliferation [9]. Antioxidant activities were examined by assessing ROS generation in different thyroid cancer cells influenced by polyphenols. In these cell lines, the phenolic compound treatment led to ROS level reduction. Thereupon, antioxidant molecules are revealed as appropriate candidates for refractory DTC including such mutations [10].

**Figure 1:**
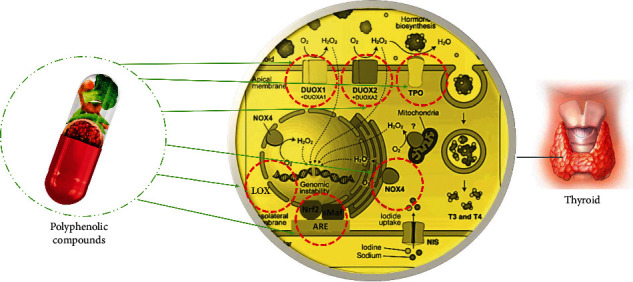
Targeting the enzymes related to oxidative stress-induced thyroid cancer by polyphenolic compounds.

Plant-derived ingredients as functional foods, unlike chemical anticancer drugs which exert high levels of toxicity, represent the advantages of low toxicity and useful tools in preventing cancer. Indeed, the results from in vitro studies could encourage the search for novel strategies in the future. Thus, they have attracted interest in exploring safer anticancer drugs with high satisfaction [11]. Polyphenols have a wide range of pharmacological properties, including the potential to serve as anticancer agents [12]. Numerous chemotherapies have failed as a result of adverse effects, drug resistance, and the target specificity of some medicines. There is growing interest in creating medications that address these issues through the use of natural chemicals that can reach several targets with little side effects and are effective against several forms of cancer [13]. The primary reason that cancer treatment does not respond because of drug resistance develops over the course of the disease. Polyphenols have mostly been shown to inhibit enzymes and act as ligands for signal transduction receptors in vitro. Thus, the point interactions of polyphenols with proteins as well as their antioxidant characteristics are critical factors that lead to their potent health benefits. Additionally, certain effects are likely the result of a combination of radical scavenging and contact with the activities of enzymes. A phenolic nucleus is a structure that is well adapted for molecular interactions with proteins [14, 15]. The cross-talk between polyphenols and important enzymes involved in neoplastic cells and oxidative processes must be completely understood, as it will provide fresh insight into the treatment of thyroid problems. Polyphenols have the ability to influence a variety of biological events occurring in thyroid cancer. The purpose of this article is to discuss the effect of polyphenolic compounds on oxidative stress-induced thyroid cancer. Follicular cells require continuous physiological generation of H_2_O_2_ as the starter of a set of oxidation processes necessary for thyroid hormone synthesis, culminating in the organification of iodide by iodination of Tg. As a result, it is unsurprising that thyrocytes' defense against oxidative stress is so critical. Nonetheless, in comparison to other tissues, notably the thyroid, there is a dearth of evidence on the role of antioxidant systems. Additionally, we will discuss the possibility of using polyphenols as modulators of oxidative stress-related enzymes. We focused on naturally occurring redox-active chemicals that may have an effect on the thyroid gland and implicate enzymes involved in the oxidative pathway. The purpose of this research was to conduct a review of the literature on natural chemicals having the potential to impact the physiology or pathology of the thyroid gland, with a particular emphasis on those known to modify enzyme activity as a mechanism of antioxidant response (Figure 1). Figure 1 shows the summary and main aim of this study.

## 2. Oxidative Stress and Thyroid Cancer

It is generally established that reactive oxygen species (ROS) have a role in a variety of inflammatory and chronic disorders, including cancer. Under normal conditions, the redox state of the cells is maintained by a balance between ROS generation and antioxidant detention [16–18]. While ROS production is required for the host's innate immune response to external pathogens such as bacteria and viruses, its increased production results in an imbalance in the cellular redox potential, leading in signaling cascade changes. With regard to the type and nature of prooxidative stressors or changes in autonomous cellular circumstances or cell types, disruptions in ROS production from any source (i.e., regulated by diverse cellular compartments) have been connected with neoplasia [12, 19]. The sources of ROS in tumors may be suggested as novel targets for antitumorigenic therapies. According to recent studies, oxidative reactions have interdependence with thyroid cancer occurrence [20, 21]. It might be assumed as an identification marker that separates thyroid cancer from benign thyroid tumors. Collected data around thyroid cancers have shown a remarkable increase in serum oxidant levels and a decrease in antioxidant levels in cancer cases compared to healthy ones [7, 22].

Alterations occurring in oxidative stress including the high production of ROS can trigger the progression of thyroid malignancies. This might be a marker to identify thyroid cancer from various kinds of benign thyroid tumors. Studies have investigated the remarkable enhancement of oxidant levels of sera in TC patients and, on the other side, the diminution of antioxidant levels in TC patients compared to healthy controls [7]. The concentration of oxidized lipid-derived aldehyde named malondialdehyde increases in TC patients' blood [23, 24]; conversely, a reduction in the antioxidative capacity of whole blood can be detected. Indeed, studies showed decreased levels of catalase expression in sera of TC patients. The most serious hallmark step of carcinogenesis is oxidative stress inducing cell DNA damage [25]. Due to the low redox potential of guanine, oxidation is thought to be a useful indication of DNA damage during carcinogenesis. Oxidation of DNA can be occurred as well by H_2_O_2_ itself and H_2_O_2_-derived hydroxyl radicals (OH•-) which can inert towards DNA and involve DNA oxidation. Under stressful conditions, an excess of ROS liberates “free iron” from iron-containing molecules and forms H_2_O_2_-derived hydroxyl radicals. These radicals are closely related due to Fenton reaction's involvement of redox-active metals such as iron. Experiments indicate that the phosphorylated form of the histone H2AX and a 53BP1 marker are both significant indicators of DNA lesions and DNA damage responses [26]. As mentioned, the duty of hydrogen peroxide in the process of TH synthesis is finely described, but its exact molecular mechanisms in pathological contexts contain open questions.

## 3. Antioxidant Properties of Polyphenols on Thyroid Cancer

Pathological conditions occurring in the thyroid are linked with oxidative stress. This event is the reason for mainly excessive generation of reactive oxygen species, not eliminated by normal antioxidant agents in the body. These pathways may be amplified by antioxidative materials, such as phytochemicals, especially phenolic ones [27]. It is accepted that plants are a significant source of secondary metabolites, including polyphenols with significant biological antioxidative effects [28]. Among these, phenolic compounds are the most abundant class of natural antioxidants, with both direct and indirect antioxidant activity that contributes to the reduction of oxidative stress [29]. Approximately more than half of the anticancer drugs that are considered helpful and effective for cancers originated from herbal, marine, and microorganism sources [30]. The anticancer therapeutic efficacy of phytochemical compounds has been detected widely and has demonstrated interesting results [31]. Phenolic compounds are a rich source of novel prospective antithyroid cancer agents and have been shown to inhibit cell viability in several kinds of thyroid cancer cells. Although abundant preclinical studies data are existing, the clinical evidence for the potential efficacy of these herbal compounds on thyroid cancer is scarce and limited [32].

Chronic uncontrolled inflammation results in the continual creation of harmful oxygen species can induce DNA damage, genomic changes, and tumor formation. Additionally, an endless supply of inflammatory mediators such as IFN, TNF, and IL-1/IL-6 and proangiogenic growth factors such as cytokines and VEGF are generated. These molecules promote tumor neovascularization by nourishing the growing tumor with blood supply. Ultimately, inflammation elevates tumor metastasis via the excessive production of extracellular matrix-degrading enzymes including MMPs. Polyphenols block tumor cell proliferation through inhibition of ROS production and suppression of COX-2, 5-LOX, and xanthine oxidase which possess the key catalysts for tumor progression [33]. Isoflavones also have the capability of inducing cancer cell apoptosis by blocking ROS formation, inhibition of DNA topoisomerase I/II activity, suppression of Mcl-1 protein and NF-*κ*B, and activation of endonuclease [34]. Polyphenols can help to protect from oxidative stress by inhibiting inflammatory responses via the MAPK signaling pathway [29, 35].

Vital biomolecules including lipids, proteins, and DNA can be irreversibly destroyed by super reactive intermediates such as ROS which is the primary external component of oxidative stress [36, 37]. Polyphenols operate as potent antioxidants by donating electrons or hydrogen to reactive oxygen, nitrogen, and chlorine species, therefore inhibiting the formation of unstable radicals [38]. Polyphenols are effective radical scavengers and hydroperoxide neutralizers. Additionally, they have the ability to serve as metal chelators, converting metal prooxidants into stable compounds. The antioxidant activity of a phenolic compound is essentially determined by the structure of the molecule, as well as the number and location of OH branches. Additionally, a larger concentration of hydroxyl aromatic rings in flavonoids has been associated with improved antioxidant activity. It is critical to understand that when a phenolic molecule loses an electron or serves as a reducing agent, the molecule transforms to a radical, although a reasonably stable one; its oxidized intermediates appear to become prooxidants. Thus, polyphenols can be a double-edged sword; on the one hand, they are effective antioxidants against excessive oxidative stress, such as ROS, and thus helpful to health; on the other hand, when eaten in large amounts, they can exhibit prooxidant activity [39]. Antioxidant/prooxidant effects of natural antioxidants depend on various factors. In the case of polyphenols, it depends on their structures as orthodi-/trihydroxylated compounds, which are more capable of prooxidant activity than their monohydroxylated polyphenolic analogs. This prooxidant property is related to transition of metal ions, such as Cu2+ and Fe3+. The prooxidant activity is a double-edged sword. In some cases, prooxidant activity results in DNA damage and mutagenesis or however represents a role in their selective cytotoxicity toward cancer cells due to extent copper. Orthodi-/trimethoxylated phenol B ring flavonoids do not have significant prooxidant activity compared with their counterparts with orthodi-/trihydroxylated phenol B ring flavonoids [40].

ORAC, FRAP, DPPH, and PCL are all methodologies for determining the antioxidant activity of polyphenols in chemical models and phenolic extracts. These approaches were developed as rapid screening tools, but none of them is applicable to the real physiology of the body [38]. Due to the poor bioavailability of polyphenols, the physiological concentration of polyphenols is too low for a significant number of chemical-based methods. The current generation of cell-based antioxidant assays (CAA) is one step closer to physiological conditions since it is conducted at physiologically appropriate phenolic and other phytochemical antioxidant concentrations [41].

Halliwell and Gutteridge [42] suggested a definition for an antioxidant as the molecular process remains unknown. Numerous flavonoids have much higher antioxidant capacities than several vitamins. Flavonoids can protect against oxidative stress caused by unstable radicals through the following mechanisms: (1) direct scavenging of ROS, (2) activation of antioxidant enzymes [12], (3) metal chelating activity, (4) reduction of *α*-tocopheryl radicals [43], (5) blocking of oxidases [44], (6) alleviation of oxidative stress occurred by NO, (7) enhancing the uric acid levels [45], and (8) increasing the antioxidant properties of low molecular antioxidants [46].

Flavonoids are direct scavengers of free radicals via hydrogen atom donation. The varied arrangement of functional groups on flavonoids' core structures, configurations, and total amount of hydroxyl groups may explain their in vitro antioxidant activity [44]. The main factor which is the positive cause of free radical scavenging rate for flavonoids is B ring hydroxyl configuration, while the ring A or C influence on the scavenging rate is low [47–49]. Polymerization of flavonoid monomers could be the reason for enhanced *in vitro* antioxidant properties. For example, polymers of catechins have more antioxidant activity than monomers. Due to the high amount of hydroxyl groups in their architectures, these compounds are exceptional in vitro antioxidants. The antioxidant activity of proanthocyanidins is due on their oligomer chain length and the kind of reactive oxygen species (ROS) involved in the reaction [50]. On the other way, depletion in the antioxidative level of flavonoids might be owing to the glycosylation of these compounds compared to the aglycone forms [51–53]. Quercetin glycosylation substantially decreases its superoxide and hypochlorite scavenging ability [54] and also reduction of *Fe^3+^* to *Fe^2+^*[55].

As an example of a free radical scavenger among “natural” antioxidant compounds, olive tree phenolic component oleuropein (OLE) is a phenolic product of virgin olive oil that has been shown to have antioxidant and antiproliferative properties in several preclinical models of cancer illness [56, 57]. OLE has been shown to decrease the viability of thyroid cancer cells at micromolar doses compared to those employed in other tumor cellular models [58, 59]. The antioxidative effects of OLE and its peracetylated derivative against two thyroid cancer cell lines, BCPAP and TPC-1, were examined in a study by Bulotta et al. They demonstrated that the phenolic compound of virgin olive oil had an inhibitory effect on thyroid tumor cell growth. Also, they reported low levels of phosphorylated Akt and ERK and H_2_O_2_-induced ROS. Peracetylated derivatives of OLE could inhibit cell proliferation and act as an antioxidant stronger than OLE. This effect is the result of chemical modification that led to improved permeability due to extensive deacetylation of hydroxytyrosol acetate. More investigations will expose the strong application of modern targeted therapies for thyroid malignancies [10].

**Figure 2:**
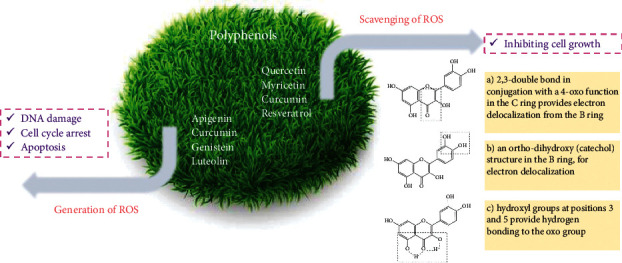
Role of polyphenols on scavenging or generation of ROS. The main structural features of polyphenols required for efficient radical scavenging include the following: (a) 2,3-double bond in conjugation with a 4-oxo function in the C ring provides electron delocalization from the B ring. (b) An orthodihydroxy (catechol) structure in the B ring, for electron delocalization. (c) Hydroxyl groups at positions 3 and 5 provide hydrogen bonding to the oxo group.

Some polyphenol treatment causes DNA damage and cell death in thyroid cancer cells via inducing ROS accumulation. For instance, a PTC cell line, i.e., BCPAP, was affected with apigenin for evaluation of its influence on cell growth. Apigenin had the ability for decreasing a protein coding gene, i.e., cell division cycle 25C expression. This process led to cell cycle block and accumulation of free radicals. Apigenin also led to the stimulation of DNA injury in cells. In summary, apigenin diminished cell viability by inducing ROS generation, leading to DNA injury and cell cycle arrest [60, 61]. Another example of the anticarcinogenic effect of polyphenols via ROS generation is curcumin. After curcumin treatment, ROS was accumulated and the apoptosis of the K1 PTC cell line induced. ROS generation also led to the crumble of MMP and high intracellular Ca2+ influx, resulted in apoptosis and prevention of thyroid cancer cells migration (Figure 2) [62].

Interaction with antioxidant enzymes is the other conceivable mechanism for flavonoids. Flavonoids have the potential of activating enzymes such as phase II detoxifying enzymes including glutathione S-transferase, NAD(P)H-quinone oxidoreductase, and UDP-glucuronosyl transferase with antioxidative activities. The mentioned enzymes are the key enzymes for defense against oxidative stress. Polyphenols containing an OH group in the ring C are the significant inducers of antioxidative enzymes, while those lacking this OH group pose low luciferase induction. Specific flavonoids are known to chelate Fe and Cu, therefore removing causative agents for the generation of ROS. Flavonoids are hydrogen donors to *α*-tocopheryl radical, which is a powerful prooxidant. Flavonoids can inhibit cyclooxygenase, microsomal succinoxidase, lipoxygenase, NADH oxidase, and NO production [63, 64]. Several researches have demonstrated the potential role of polyphenols in protection against oxidative stress. Of note, these studies did not centralize on thyroid tissue or thyroid cell lines; therefore, further study is required to evaluate if the potential of activating antioxidant enzymes and reducing *α*-tocopheryl radicals are also applicable for the thyroid samples. According to Ruggeri et al., the relationship between dietary habitat and redox homeostasis in thyroid autoimmunity was investigated. Their study provides the first evidence of protective potential of the Mediterranean diet against thyroid autoimmune diseases. They suggested that animal foods seem to be relevant to the development of HT associated with lower levels of the antioxidants such as GPx, GR, and TRxR but higher levels of the oxidants AGEs and AOPPs. On the other hand, plant foods containing high amounts of antioxidants may be protective against thyroid autoimmunity. They reported that the Mediterranean diet can lead to reduced risk of thyroid autoimmunity [65].

While the precise antioxidative properties of polyphenols can be determined in *in vitro*, the bioactive forms of flavonoids found in plants are not identical in *in vivo* condition (i.e., flavonoid glycosides). They demonstrated a range of absorbance properties due to their extensive biotransformation in the small intestine and hepatic metabolism. Significant phase I deglycosylation of flavonoid glycosides and phase II glucuronidation, sulfuration, and O-methylation of the resulting aglycones are involved in these metabolic changes [15, 66]. Indeed, colonic microflora takes part in the cleavage of polyphenols. Bacterial-based enzymes participate in the pathways of sulphates, glucuronides, and glycosides, demethylation, dehydroxilation, reduction of double bonds, and decarboxylation of some polyphenols [67]. Additionally, flavonoids may participate in oxidative metabolism, P450-related metabolism, and thiol conjugation [68, 69]. Certain kinds of polyphenols' O-methylated and glucuronidated derivatives have less antioxidant potential than the aglycone. Glucuronidation and O-methylation of polyphenols also decrease their inhibitory action on peroxynitrite-induced tyrosine nitration in contrast with the aglycones. There is also confirmation about the power of polyphenols in reducing *Fe^3+^* to *Fe^2+^*. It was significantly decreased by methylation of the ring B's 3′ and 4′ locations [70–72]. These findings indicate that the antioxidant power of flavonoids is under the performance of metabolic modifications. Importantly, the majority of the investigations into the prooxidant activity of natural antioxidants have been reported on the basis of *in vitro* conditions. Indeed, it appears as though the antioxidant or prooxidant properties of polyphenols are mostly dependent on their content. The understanding of which flavonoids act as anti- or prooxidant agents in *in vivo* is currently limited, and this area deserves more research [73].

## 4. The Role of NOX and DUOX in Thyroid Oxidative Stress and Modulation of their Activities by Polyphenols

**Figure 3:**
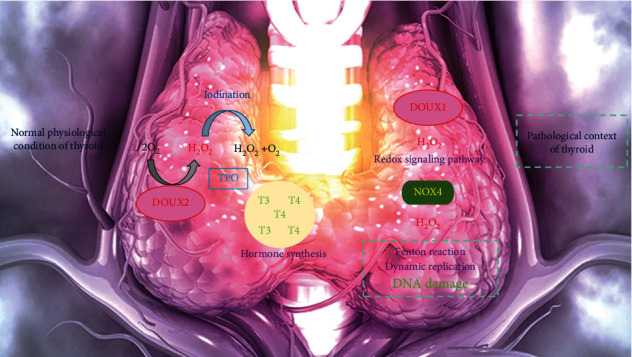
Comparison of H_2_O_2_ role between normal physiological condition of thyroid and pathological context of thyroid. Under stress conditions, H_2_O_2_ behaves as a potent oxidant. Moreover, H_2_O_2_ has the potency of causing high levels of oxidative DNA damage occurred in thyroid cells. In this condition, DUOX1 is the producer of H_2_O_2_. The physiological function of thyrocytes is thyroid hormone biosynthesis and DUOX2 is the main provider of H_2_O_2_ in this process. TPO is an H_2_O_2_-consuming enzyme which protects DUOX2 from inhibition by H_2_O_2_.

One of the specific enzyme families with the primary function yield of producing ROS is named NADPH oxidases (NOX) [74]. The NOX enzymes include members of NOX1-5 and DUOX1-2. The NOX physiological activities are contributed to defense system, cellular signaling, and cell differentiation. Indeed, extensive NOX activity is linked to pathological processes, including neurodegeneration, organ failure, and cancer. NOX4, DUOX1, and DUOX2 are informed to be expressed in thyroid tumor cells. Tumor cells generate high amounts of free radicals via NADPH oxidases to carry out cancer cell growth, through regulation of growth factors and survival factors [75]. Recent evidence informed that ROS originated from NOX4 function is the starter of thyroid carcinogenesis. Although NOX4 seems to have a critical function in thyroid tumorigenesis, the pathways contributing to NOX4 association with thyroid cancer need to be figured out in depth. The main enzymes of the oxidation steps in TH biosynthesis are thought to be TPO and DUOX2. The vital substrate of TPO as a key enzyme in the TH synthesis process is H_2_O_2_. Indeed, under stress conditions, it behaves as a potent oxidant. Moreover, H_2_O_2_ has the potency of causing the excess amounts of oxidative DNA damage that existed in follicular cells. The damages include strand breakages, DNA base lesions, chromosome instability, and association with tumor progression. The normal physiological duty of thyrocytes is TH biosynthesis, and DUOX2 is the great provider of hydrogen peroxidase in this process. TPO is accepted as an H_2_O_2_-consuming enzyme that protects DUOX2 from inhibition by H_2_O_2_ [76] (Figure 3). The iodination and organification are done by TPO, while supplying the H_2_O_2_ for TPO is the task of DUOX2 which delivers electrons in these reactions. Any mutations of DUOX1/2 and NOX4 enzymes are attractive subjects owing to their unregulated ROS production. NOX enzymes are recognized as valuable pharmacological targets in a variety of disease treatment [77–79]. Future studies will represent a better cognition of DUOX and NOX enzyme tasks in thyroid physiopathology. This awareness will authorize appreciation of DUOX/NOX enzymes for being novel markers and pharmacological targets to improve the diagnosis of thyroid pathologies and more personalized therapies [80].

Focusing on enzymatic ROS sources by the effect of herbal compounds, without interfering with normal oxidant/antioxidant state, should be a valuable idea. The duty of NADPH oxidase enzymes in physiological and pathological conditions and their suppressors originated from herbal compounds is represented recently. The utilization of natural antioxidants represents a new achievement to NOX repression. It was established that polyphenols not only represent free radical scavenging but also lessen NOX enzyme activity. According to scientific reports, Nox4 is known to be a potent oncotarget. Cell proliferation was inhibited due to NOX4 knockdown in papillary thyroid cancer cell lines. NOX can regulate glycolysis through the mROS-HIF1*α* pathway and maintains HIF1*α* stability, thereby mediating proliferation in thyroid carcinomas [81]. It has demonstrated that blueberry-derived polyphenols can disrupt NOX assembly in lipid rafts. Resveratrol can inhibit high glucose induced oxidative stress in endothelial cells by blocking NF-*κ*B/NADPH oxidase/ROS pathway. In the case of thyroid cancer, further investigations about the main molecular repressors of NADPH oxidase are required. More knowledge is essential to understand the mechanism of regulation and targeting different subcellular compartments due to NOX enzyme complexity and having different specific targets within each isoform [22, 75, 82]. Due to our increased understanding of oxidative stress events in thyroid cancer, future therapeutics utilizing NOX4 inhibitors, either alone or in combination with other agents, must be studied.

## 5. The Role of TPO and LOX in Thyroid Oxidative Stress and Modulation of their Activities by Polyphenols

Thyroid peroxidase (TPO) is a membrane bound protein necessary for the synthesis of thyroid hormones. Protein kinase A is a factor that phosphorylates and activates this enzyme. TPO has three functions: (1) oxidation of iodide to iodine, (2) organification of tyrosine residues of thyroglobulin protein with iodine, and (3) coupling reactions led to T3 and T4 production. It accelerates the iodination and organification of tyrosyl residues to thyroglobulin resulting in TH biosynthesis. The biological reason for a typical TPO expression in thyroid tumors is obscure, but the progressive reduction of TPO levels together with an increase in cell density suggests that it is linked with the proliferation process. The suggestion of TPO as an effective diagnostic marker for separating benign from malignant disease appears not to be a good idea. TPO level is significantly lower in thyroid cancers in comparison with benign conditions or normal tissues. In the expression and activity of TPO, 55–70% is observed in differentiated thyroid carcinoma, but in the case of anaplastic tumors, they have nonexistent TPO expression [83]. TPO inhibitors are found to be powerful therapeutic factors for hyperthyroidism. The main purpose for targeting the polyphenols as inhibitors was their existence in food sources and illustration of high antioxidant capacity. The antioxidant activity is the senior feature of polyphenols, which represents anticarcinogenic effects [28].

These antioxidant systems contain a diverse array of vitamins, minerals, and other small substances. Among them, selenium is an essential micronutrient with proposed role in the protection of DNA, proteins, and lipids from oxidative damage and in the homeostasis of the thyroid gland. Selenium exerts protective effects against oxidative stress in human thyrocytes. Also, it can reduce H_2_O_2_-induced apoptosis by modulation of Bcl-2 and BAX expression and inhibition of caspase-3 activity [84, 85]. It also acts as an antioxidant, and selenium deficiency has been linked to a variety of disorders, including cancer, but the specific mechanism is unknown. Selenium has been linked in the development of autoimmune thyroiditis due to its antioxidant properties. Numerous studies have demonstrated that selenium administration can help individuals with autoimmune thyroiditis reduce thyroid autoantibodies. Selenium therapy has been shown to lower TPO antibody titers in euthyroid individuals with autoimmune thyroiditis by strengthening the defense against oxidative stress and scavenging free radicals [86].

Selenoproteins, a family of selenium-containing proteins, are crucial for maintaining a healthy balance of oxidant and antioxidant species. Selenoproteins such as glutathione peroxidases (GPxs) and thioredoxin reductases (TrxRs) play a role in antioxidant defense. Seleno-enzymes, such as glutathione peroxidase, were detected in high amounts in the blood of individuals with thyroid cancer. Given the association between increased oxidative stress and thyroid cancer, it has been postulated that the impairment of selenoprotein expression may be a critical aspect in elucidating the link between thyroid cancer and oxidative stress [87]. GPX1 can decrease prosurvival signaling via the AKT pathway and suppress the production of proinflammatory mediators produced from COX2. The cumulative effect of GPxs expression on carcinogenesis, on the other hand, is unclear and appears to be tissue- or context-specific [88]. The absence of adequate laboratory evidence in the interrelationships between Se status and thyroid function has hampered the clarification of the underlying molecular mechanisms and antitumor effects of the different Se compounds. The role of selenium in redox regulation and its dependent pathways are promising anticancer strategies which are now under investigation by different groups [89].

The result of phytochemicals efficacy on TPO activity has been detected rarely. The first data reported from a study was performed by Rosales-Martínez et al., in which the inhibitory influence of peanut seed coat extract rich in dietary flavonoids was evaluated on the TPO activity [90, 91] Another study examined the inhibitory effect of green tea extract (high in polyphenols) on TPO activity. The majority of the polyphenols in tea extracts were shown to inhibit TPO in a time- and dose-dependent way [28, 90]. Apigenin, chrysin, vitexin, and baicalein all have the capacity to block TPO, the enzyme required for thyroid hormone (TH) production, resulting in hypothyroidism. As discussed previously, TPO participates in oxidation processes. When polyphenols are used to inhibit TPO activity, TH production is decreased. As a result, when polyphenolic chemicals are eaten in high amounts, an elevation in TSH may result in goiter. Quercetin, fisetin, morin, kaempferol, myricetin, rutin, naringenin, and naringin can all block tyrosine iodination through TPO with varying degrees of effectiveness. By attaching reactive radicals to catalytic amino acid radicals on TPO component II, kaempferol, quercetin, naringenin, and fisetin therapies can block TPO. Myricetin and naringin have an interaction with both TPO compound I and compound II. While tyrosine iodination can be blocked by the isoflavone biochanin A as a substrate for iodination, biochanin A's increased affinity for the enzymatic iodinating TPO species initially results in an ineffective barrier. In vitro, isoflavones derived from soybeans, such as genistein and daidzein, at quantities comparable to those seen in the blood of patients ingesting soy derivatives, are capable of inactivating TPO and inhibiting iodine organification [92].

Habza-Kowalska et al., for the goal of explaining the antiradical potential of some plant extract, evaluated the influence of catechin, kaempferol, apigenin, and sinapinic acid treatments on TPO and LOX enzyme activity. They focused on the antioxidant levels of both pure polyphenols and ethanolic extracts by the ABTS method. The ABTS is the most commonly used antioxidant assay to assess the radical scavenging power of extracts. According to this test result, sinapinic acid and catechin were discovered to have the highest antiradical activity, and on the other side, the lowest activity was observed for apigenin. Polyphenols inhibited the TPO and LOX enzyme activity concomitantly. The antioxidant activities of tested plant extracts were significantly lower than the pure compounds. The polyphenols displayed various manners of inhibition. Sinapinic acid, catechin, and kaempferol were competitive TPO inhibitors, while apigenin was an uncompetitive TPO inhibitor. The highest radical scavengers belonged to sinapinic acid, catechin, and compounds derived from mustard and green coffee [93].

Lipoxygenase (LOX) is a lipid-peroxidizing enzyme that catalyzes the peroxidation of arachidonic acid and implicated in the pathogenesis of cancers. LOX can promote cancer cell survival and enhance the metastasis process. Therefore, the application of LOX inhibitors could be important in cancer managements. LOX is a key enzyme by activity of inducing oxidative stress to produce ROS ([94]; C. [95]). ROS activity can lead to cell death. The thyroid gland takes part in the autoregulation of the redox balance in normal physiological conditions. High levels of ROS activity can unsettle this balance, which can influence thyroid enzyme activity. The mechanism is yet obscure, and some investigations should be undertaken to prospect this event [96]. Inflammation and oxidative stress are closely connected pathways. Thyroid hormones act as antioxidant balancers, while both hyperthyroidism and hypothyroidism have been determined to be linked with oxidative stress [93, 97]. Plants are major sources of antioxidant polyphenols with proven biological effects. Habza-Kowalska et al. estimated the TPO inhibition manner by chlorogenic acid, rosmarinic acid, rutin, and quercetin. Obtained data indicated that all the polyphenols in this test inhibited TPO and cleansed free radicals (Table 1). Rutin and rosmarinic acid had competitive inhibition, quercetin had uncompetitive inhibition since Km and Vmax were influenced, and chlorogenic acid represented a noncompetitive inhibition effect where Vmax, but not Km, was influenced. According to the ABTS method, all the polyphenols of this assay blocked TPO activity, but with totally diverse potencies: rosmarinic acid > rutin > quercetin > chlorogenic acid. As the report of the isobolographic analysis, rutin and chlorogenic acid represented synergistic activity, whereas chlorogenic acid and rosmarinic acid were antagonists like quercetin with chlorogenic acid and rosmarinic acid with quercetin. The IC50 values for these polyphenols in different studies are different which can be associated with the differences in the methodology, solvents, and the source of TPO. For example, the IC50 values ranged from 0.004 mM to 1.44 mM in Habza-Kowalska et al.'s study, whereas in the other study, data showed that the IC50 value was different with TPO [28].

## 6. The Role of Nrf2 in the Thyroidal Antioxidant Response and Modulation of Its Activity by Polyphenols

**Figure 4:**
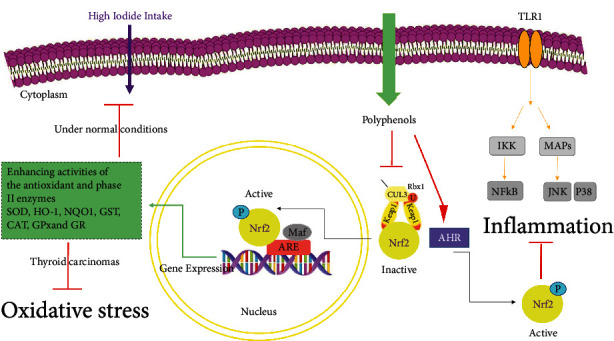
Antioxidative and anti-inflammatory effects of polyphenols in relation to Nrf2 pathway. The molecular mechanisms of activation of the Keap1-Nrf2 system by polyphenolic compounds are shown in this figure. Under normal condition, the excess uptake of iodide through the cell membrane was inhibited via Nrf2 activation, and on the other hand, in thyroid cancer condition, oxidative stress was inhibited via antioxidant enzymes.

The transcription factor nuclear factor erythroid 2-related factor regulates the expression of these antioxidant enzymes (Nrf2). At the cellular level, ROS can activate this enzyme. Nrf2 translocates into the nucleus and controls the transcription of many types of genes encoding the aforementioned antioxidant enzymes via antioxidant-responsive elements (ARE). Thyrocytes, it is considered, require extra protection against oxidative stress. While a low level of oxidative load is vital for thyrocytes' normal proliferation and function during thyroid hormone synthesis [98, 99], extra thyroidal oxidative stress can lead to pathological contexts such as goitrogenesis, iodine deficiency, and cancer. The thyroid's antioxidant defenses will be triggered in this condition [25, 100]. The Nrf2 signaling pathway plays a critical role in the thyroid glands antioxidant defenses. Nrf2 is a transcription factor that affects the expression of TrxR1 and Gpx2 [100], which are confirmed to modulate homeostasis by alleviating oxidative damages in thyroid cells [101]. Indeed, Nrf2 defends the thyroid cells from oxidative injuries induced by extra iodide uptake. Nrf2 balances the thyroid gland antioxidant defenses with Tg synthesis and iodination [92]. Therefore, Nrf2 represents pleiotropic functions such as combining cell defense systems to iodination and the TH biosynthesis [100]. Nrf2 prevents excessive Tg iodination by lowering ROS levels via a positive regulation of antioxidant gene expression under normal physiological contexts. In the presence of iodide overload, Nrf2's protection against oxidative stress is even more critical in defense against excessive iodine consumption. Nrf2 initiates a transcriptional cascade that suppresses ROS generation, therefore protecting biomolecules from oxidative damage in follicular cells [102]. Due to the protective and physiological activities of Nrf2 signaling in normal thyroid, it has been documented that it is activated in thyroid carcinomas, where it plays a role in oxidative stress resistance and protection (Figure 4) [103, 104].

According to Kansanen et al., the Nrf2-Keap1 pathway regulates redox homeostasis and detoxification [105]. Organoselenium compounds with thiol peroxidase activity are frequently referred to as glutathione peroxidase mimetic. In addition, they may increase ROS generation and oxidation/depletion of cellular thiols, including the Nrf2-Keap1 complex. Selective oxidative and covalent modifications of selected cysteine residues by Se organic peroxidases affect this enzyme protein's GSH-dependent detoxification function, which is critical in therapeutic protocols against drug-resistant tumors, as well as its redox sensing and signaling functions via cellular stress and death pathways. In order to advance the development of these compounds for cancer prevention and drug-resistant tumor treatment, these aspects are examined extensively. Cancer therapy may benefit from Se compounds that affect the Nrf2-GSH axis of the GSTP molecular interaction. The study of the therapeutic mechanism of Se compounds could lead to new polyphenol-polyphenol combinations in the treatment of drug-resistant cancers [106, 107]. Polyphenols can also trigger nuclear translocation of Nrf2 to induce expression of the antioxidant defense genes through interaction with cytosolic AhR [108]. Flavonols and isoflavones have shown agonistic effects for modulation of AhR-mediated signaling [109]. For instance, luteolin, quercetin, chrysin, and apigenin have been accepted as AhR agonistic regulators. By enhancing the actions of enzymes such as CAT, SOD, GR, and GPx, polyphenols have the capacity to restore redox equilibrium and avoid inflammation. Polyphenols' impacts on AhR and Nrf2 signaling pathways are critical molecular processes for promoting endogenous antioxidant defense systems based on SOD, CAT, GPx, and GR. These mechanisms underpinning polyphenols' actions resulted in the restoration of cellular redox homeostasis (Figure 4) [29]. Nrf2 is a key regulator of antioxidant cellular responses and a target of phytochemicals, which have been shown to protect against oxidative stress-related illnesses [100]. As a result of this, these phytochemicals are referred to as indirect antioxidants. The majority of Nrf2's targets are cytoprotective and antioxidant enzymes. The sulforaphane-rich broccoli sprout extracts are a well-designed Nrf2 activator that is found in its precursor form in phytochemicals [110, 111]. Nrf2 is stimulated by a variety of natural compounds, not just sulforaphane. In general, natural chemicals that can react directly with Keap1's cysteines have the ability to activate the Nrf2 pathway [112]. Numerous researches have examined the effect of phytochemical-mediated Nrf2 regulation on the thyroid gland and associated diseases.

The influence of phytochemicals on thyroid function and the possible underlying modification of the Nrf2 pathway, a critical mediator of antioxidant, cytoprotective, and other actions in cells, have been examined infrequently. Numerous studies have proven the critical function of polyphenols in Nrf2 activation against oxidative damage, inflammation, and cancer. Although these studies did not explicitly examine thyroid cells, more study is needed to determine whether the effects mentioned on the Nrf2 pathway are also applicable to thyroid cells. Among phenolic compounds, EGCG, curcumin, and catechins exert antioxidative effects via the performance of the antioxidant enzymes which are under the regulation of Nrf2. Curcumin's ability to induce Nrf2 activity is dose- and time-dependent, resulting in the stimulation of HO-1 expression [113, 114]. According to Paredes-Gonzalez et al., luteolin and apigenin both activate Nrf2 and downstream genes and protect against oxidative stress through induction of HO-1, glutamate cysteine ligase, and suppression of cPLA2 and LOX via the Nrf2 pathway [115]. Biochanin A displayed antioxidative [116] and hepatoprotective activity via stimulation of Nrf2 translocation and high expression of HO-1 [117]. According to a study, hesperidin caused an elevation in the myocardial antioxidative condition via lessening lipid peroxidation and protein oxidation, enhancing GSH levels and the Nrf2 pathway, which led to higher expression of antioxidant enzymes [118]. Morin had protective activity in resistance to oxidative stress pathways. Following H_2_O_2_ exposure to C2C12 myoblasts, morin significantly prevented ROS generation and DNA damage via upregulation of Nrf2-dependent HO-1 expression through the ERK signaling pathway [119]. In the case of myricetin, proinflammatory molecules were inhibited through the suppression of STAT1 and NF-*κ*B by induction of HO-1 expression mediated by Nrf2 in LPS stimulated macrophages [73].

Du et al. investigated the molecular basis for the effects of Pinelliae rhizome on the human papillary thyroid cancer cell lines using Nrf2 siRNA (siNrf2). Treatment of transfected cells with Pinelliae rhizome indicated that silencing of Nrf2 can decrease the apoptosis rate. The obtained data informed that the survival rates were low in Nrf2-knockout mice compared with wild-type, revealing Nrf2 importance as a starter of protective antioxidant response [11]. According to Ginwala et al., experiments using the p62/Keap1/Nrf2 pathway demonstrated that Nrf2 was activated concurrently with autophagy following apigenin administration, supporting the link between autophagy and oxidative stress. It is informed that apigenin has the ability for stimulation of Beclin-1 aggregation, transformation of LC3 protein, degradation of p62, existence of autophagosomes, and formation of AVO. These consequences declare that apigenin concurrently induced cell death and autophagy. In response to ROS and oxidative stress, autophagy may be triggered. Autophagy, being a powerful cleanser within the cell, is critical for eliminating oxidatively damaged organelles and so serves as a major line of defense against oxidative stress damage. Indeed, they investigated whether apigenin promoted autophagic death via the formation of reactive oxygen species (ROS). As a result, apigenin accumulated substantial amounts of ROS intracellularly. ROS have the potential to do significant damage to biological molecules such as DNA, RNA, and proteins. Apigenin has been shown to stimulate ROS production, resulting in DNA damage and consequent cell cycle arrest. Autophagy-mediated cell death is evident in human PTC cells. Apigenin, on the basis of these data, is a chemotherapeutic factor with therapeutic significance in TC treatment. Additionally, because apigenin inhibits the autophagy route, it increases the absorption and sensitivity of ^131^I in thyroid cancer therapy, which warrants further exploration [34, 120].

It is realized that oxidative stress is closely associated with cancer evolvement. Genistein is a well-known powerful antioxidant that has been shown to protect against oxidative stress in a variety of malignant and noncancerous tissues [121]. Genistein is an isoflavone found in legumes and soy products that possesses a number of qualities that may be advantageous in the treatment of a variety of human disorders, including cancer. It has been shown to impede the development of thyroid cancer cell lines FTC-133, NPA, TPC-1, FRO, and ARO cells. Genistein can inhibit the growth of primary papillary thyroid cancer cells by exerting a protective effect against oxidative DNA damage without causing primary DNA damage [32, 122]. Genistein can cause higher nitric oxide production and induce apoptosis. Genistein intensifies NOS activity [123], which contributes NO synthesis from its substrate [121]. Activation of the Nrf2-HO-1/NQO1 pathway mediated by genistein signified great inhibitory effects on oxidative stress. After Nrf2 phosphorylation, it can activate ARE and lead to high transcription of NQO-1, HO-1, and GST genes which are regulated by Nrf2. Genistein represents a serious effect in amplifying antioxidant enzyme activity. The stimulation of antioxidant enzymes by genistein is mediated through PTEN and AMPK pathways. Genistein also reduces ROS levels by stimulating the expression of the MnSOD and CAT. For these reasons, genistein is an attractive key compound for developing novel and more effective drugs for combating malignant thyroid cancer in the coming times [124]. Curcumin represents antioxidative and anti-inflammatory activities together, therefore having a remarkable influence on the occurrence of cancer prevention strategies and applications in clinical research. Several studies evaluated the relationship between ROS level and apoptosis induced by curcumin. The pathway of curcumin-mediated apoptosis was determined to be linked with ROS production. Nrf-2 is a significant molecular switch via which curcumin may exert its health benefits. Curcumin has been shown to boost the activity of ROS scavenging enzymes. In malignant cells, ROS is a bifunctional biological molecule. It can cause DNA alterations during carcinogenesis and can promote mitochondrial apoptosis [123].

The attractive hypothesis proposed that following intracellular ROS increasing, these reactive oxygens can oxidize polyphenolic compounds to electrophilic hydroquinones and quinones. In this chemical form, herbal components can oxidize certain cysteine residues on Keap1, which binds and maintains an inactive, cytoplasmic Nrf2. After oxidation of cysteine, the bindings between Keap1 and Nrf2 cleavage and Nrf2 translocate to the nucleus for binding to EpRE which are the effectors of the antioxidant enzyme gene transcription. According to these attractive findings, we can demonstrate that low concentrations of polyphenols, i.e., concentrations which are not enough to act as direct antioxidant agents, following blandly oxidized, can generate nontoxic amounts of electrophilic compounds which are not harmful to cells but are necessary for triggering the Keap1-Nrf2-EpRE pathway antioxidant responses [125, 126].

## 7. The Role of LPO in Thyroid Oxidative Stress and Modulation of Its Activity by Polyphenols

**Figure 5:**
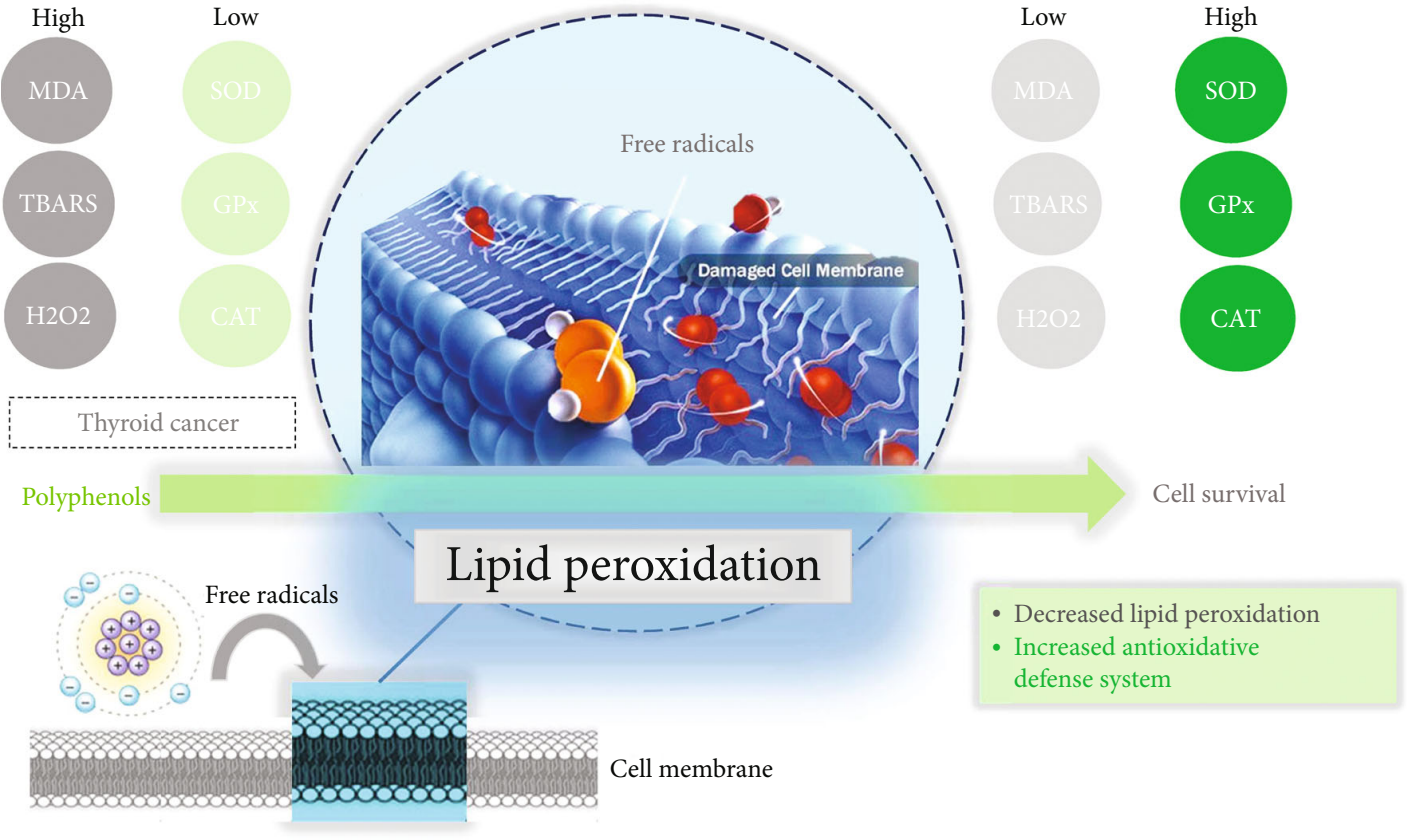
Lipid peroxidation in thyroid cancer and its modulation by polyphenol. High generations of free radicals are linked with high intensity of lipid peroxidation in patients with papillary thyroid cancer. Increased lipid peroxidation can lead to oxidative damage and direct damage to membrane lipids. Moreover, the activities of antioxidative enzymes such as SOD, CAT, and GPx were reduced in cancer tissues. By the influence of polyphenols, antioxidative enzyme activity increased and the products of LPO such as MDA, TBARS, and H_2_O_2_ decreased.

Lipid peroxidation (LPO) is known to cause membrane disturbance and modification of proteins and DNA bases which can be led to a number of diseases. As a redox signaling mediator, LPO products were seen to be important in this process. During lipid peroxidation, oxidants such as free radicals attack lipids especially PUFAs. It has long been recognized that uncontrolled oxidative stress can inflict lipid damage. Lipids are the important component of the cell that is more sensitive to free radicals effects. When membrane lipid peroxidation rates are low, cells may promote survival via antioxidative defense mechanisms; nevertheless, when membrane lipid peroxidation rates are high, cells trigger apoptotic pathways [127]. Malondialdehyde (MDA) as the product of lipid peroxidation is known to be the best marker to indicate oxidative damage in thyroid cancer tissues compared to normal tissues. Accelerated mitochondrial electron transport can be the feedback of thyroid hormone function resulting in the increased production of superoxide. Superoxide radicals can lead to the generation of many other reactive species, such as hydroxyl radicals. This event results in increased lipid peroxidation which leads to oxidative damage and membrane lipid damage. It has been reported that high generation of free radicals is linked with high intensity of lipid peroxidation in plasma/serum of patients with thyroid cancer. In this condition, antioxidative enzymes such as SOD and GPx initiate the ROS production control. Moreover, it has demonstrated that activities of SOD, CAT, and GPx were reduced in some types of thyroid cancer tissues (Figure 5). Although the expression of redox factors in different types of thyroid cancer cells is not similar. SOD1 stayed at similar levels, SOD2 expression increased in cancer, and SOD3 mRNA synthesis decreased correlating to reduced differentiation degree of thyroid tissue [128]. The higher expression of NIS, PDS, and TSHR in adolescents suggested a greater degree of differentiation of thyroid carcinomas in this age group. The opposite is also true; that is, lower expression of this thyroid-specific gene may indicate a lower grade of tumor differentiation and, therefore, a more aggressive thyroid tumor behavior in children [129]. Low expression of NIS and other genes required for iodine incorporation are known as hallmarks for patients suffering from advanced thyroid carcinoma. In these cases, refractory to radioiodine therapy is abundant [130]. Given that NIS expression is frequently decreased in malignant thyroid tissues, considerable effort has been devoted to figure out NIS modulation in thyroid cells with the hope of restoring NIS expression in thyroid cancer cells. It is interesting to note that several reagents have been shown to be possible candidates to enhance thyroidal RAI accumulation leading to high levels of NIS mRNA/protein in clinical trials [131].

The lack of balance between oxidants and antioxidants systems can be understandably observed in thyroid cancer cells. The low expression of selenium antioxidant molecules such as GPx1 and TrxR1 in papillary thyroid cancer cells suggested that oxidative stress was involved in thyroid malignancies, since free radicals were produced in excess [87]. According to Arczewska et al., GPX1 mRNA was increased in thyroid tumor tissue, but without reaching statistical significance (2.05 ± 0.27 in tumor tissue vs. 1.60 ± 0.17 in peritumoral tissue; *P* = 0.149; *N* = 84). On the other hand, GPX1 mRNA level was increased in BRAFV600E mutated cancers [132]. According to Cammarota et al., glutathione peroxidase family had different expression: GPX1 and GPX2 expression analysis showed minor increase, and GPX3, GPX5, and GPX7 expression was low in thyroid cancers, whereas GPX4 expression indicated no changes. PRDX1, PRDX2, and PRDX3 expressions downregulated in papillary and anaplastic thyroid cancers, PRDX4 mRNA expression was moderately increased in anaplastic thyroid cancer tissues, and PRDX6 expression status showed similar levels as well as normal thyroid and thyroid cancers [128]. Catalase expression was markedly decreased in anaplastic thyroid cancer as compared to normal thyroid tissue and papillary thyroid cancer (Table 2). Also, according to Cammarota et al.'s studies, redox gene expression in PC Cl3 cells transformed with PTC1 and *e1a* oncogenes suggested partial similarity between human microarray data and cell models. The expression of nox2, nox3, and nox4 in cell models showed similar increase as in thyroid cancer tissues, whereas the decreased expression of sod3, catalase, gpx5, gpx7, prdx1, prdx2, and prdx3 corresponded to decreased expression in microarray data. It is important to note that the expression of nox1, sod1, sod2, gpx1, gpx3, gpx4, gpx8, prdx4, and prdx6 in cell models was opposite to human tissues. Further, gpx6 and prdx5 that showed variable expressions in cell models were not expressed in human cancers. In FRLT5-based H-RasV12 cell model, the expression of nox4, sod2, sod3, catalase, gpx2, gpx4, and prdx6 corresponded to human tissue mRNA production. The expression of gpx1 was variable and the expression of nox1, nox2, sod1, gpx3, gpx7, prdx1, prdx2, prdx3, and prdx4 in cell model was opposite to expression in thyroid tissue. The differences between tissue microarray database and in vitro gene expression assays may be caused by presence of stromal paracrine effect influencing tissue redox balance or presence of multiple genetic aberrations in vivo [128].

The defensive system includes enzymatic reactions and nonenzymatic reactions. The enzymatic reaction consists of SOD, GSH-Px, CAT, and GST, and on the other hand, nonenzymatic one consists of VitE, carotene, VitC, Cys, His, Ser, Met, glucose, Ceruloplasmin, Transderrin, and Lactofein. Both a deficiency in antioxidant defense components and enhanced lipid peroxidation may contribute to the development of thyroid cancer. The imbalance in oxidants and antioxidants may be connected with an increased risk of thyroid cancer. This imbalance reflects the peroxidation rate in thyroid cells. The tissue antioxidant defense in thyroid cancer is rarely studied, so the comparison between the oxidant/antioxidant status of thyroid tissues and healthy controls should be described [8, 133, 134].

Effects of *Feijoa sellowiana* extracts on antioxidant defense system, lipid peroxidation, and thyroid functions in Sprague-Dawley male rats were evaluated by Keles et al., and the experimental data indicated that *F. sellowiana* extracts had remarkable inhibiting effects on lipid peroxidation. In traditional medicine, it has been demonstrated as a remedy for goiter and cancer therapy. The *F. sellowiana* extract contains polyphenols such as daidzin, genistin, daidzein, genistein, formononetin, biochanin A, and prunetin. These metabolites and additional novel components characterized for this extract can speed up the scavenging of free radicals and protect of cell membranes. In this way, blood MDA level was decreased; GSH level and plasma antioxidant activity were increased compared with control samples [135]. *Launaea procumbens* extract is useful for the treatment of hormonal disorders and oxidative stress. Polyphenols such as vitexin, orientin, rutin, hyperoside, catechin, and myricetin are available in this extract [136]. The efficacy of this extract on oxidative stress and thyroid hormonal dysfunction in the male albino rats indicated the protective effects of *Launaea procumbens* extract including reduced T3 and T4 levels and increased activities of CAT and SOD enzymes. TBARS and H_2_O_2_ decrease in samples treated with *Launaea procumbens* extract compared to control samples demonstrating that this extract contains bioactive polyphenolic compounds which play a significant role in the lipid peroxidation system [137].

## 8. Conclusion

Recently, thyroid carcinoma has rapidly increased in prevalence as the most widespread endocrine cancer in a number of countries. Although thyroid cancer has a low death rate in contrast to other cancers, the rate of disease relapse and durability has increased significantly, which is associated to the illness's increased incurability and patient morbidity and mortality rates [2, 138, 139] While conventional surgical thyroidectomy followed by adjuvant radioiodine therapy has been the mainstay of treatment for follicular thyroid cell-derived malignancy, it is frequently not curative for various kinds of thyroid cancer. Additionally, standard anticancer therapies are ineffective due to the development of radiation resistance. Recent advances in the detection of thyroid cancer's molecular etiology imply a greater commitment to the development of more effective treatment strategies for thyroid carcinoma [139–141]. This new understanding of thyroid cancer's molecular pathophysiology has created tremendous prospects for the development of novel therapeutic therapies for reducing thyroid carcinoma prevalence. Since the relation between oxidative stress and important diseases is proposed, there are still open questions in the case of thyroid cancer patients. High oxidative stress is a risk factor linked with thyroid tumorigenesis and progression and therefore special prevention is warranted [21]. In the present review, we mainly described the antioxidative activity of some polyphenols as functional foods on thyroid cancer and hoped to offer innovative therapies by discovering new, cost-effective, and safe therapeutic agents to improve public health.

Polyphenols' effect on thyroid cancer has not been well investigated in observational studies, and the data is unclear. Further scientific study is required to provide a conclusive risk assessment of polyphenols' effect on thyroid cancer. Phytotherapeutics are an excellent source of new substances that may be effective in the prevention of thyroid cancer. It has been found that phytochemicals inhibit cell growth in many kinds of thyroid cancer cells. Additionally, these natural chemicals demonstrated efficacy as an adjuvant for radioiodine treatment. There are still underlying problems about their bioavailability. Although there is some preclinical evidence for these phytochemicals' antioxidative effects on thyroid cancer, clinical evidence is rare and limited. As a result, more clinical research is necessary to elucidate the role of phytotherapeutics in the treatment or prevention of thyroid cancer [142, 143]. The plant-originated functional foods represent antioxidant or other cytoprotective activities that have exhibited favorable and helpful outcomes with the scope of cancer prevention, but at particularly high dosages or in specific combinations, it may have a detrimental effect on thyroid function [144, 145]. Still, some of the patients were unresponsive to these kinds of therapy, which was not completely void of after-effects [146, 147]. Thus, the seeking for new aspects, main accurate molecular objectives, and effective medication for the best handling of these tumors are inevitable problems. While the health benefits of natural components present in plants in trace amounts are universally accepted, caution should be exercised when using concentrated extracts that may include excessive concentrations of phytochemicals [148, 149]. According to findings, low concentrations of polyphenols, i.e., concentrations which are not enough to act as direct antioxidant agents, following blandly oxidized, can generate nontoxic amounts of electrophilic compounds that are not harmful to cells but are necessary for triggering the antioxidant responses.

Thanks to remarkable progress in recognizing the molecular pathogenesis of thyroid cancer that have been performed recently, this review discussed the potential role of polyphenols in targeting enzymes related to oxidative pathways in thyroid glands. Herein, some enzymes such as NADPH oxidases (including DUOX1, 2, and NOX4), thyroid peroxidase, lipoxygenase, lipid peroxidase, and Nrf2 have been explained. However, the *in vitro* and *in vivo* approaches of inhibition or activation of mentioned enzymes by different concentrations of polyphenols have been studied rarely in the case of focusing specifically on thyroid tissue or thyroid cell lines; we tried to represent the antioxidative activity of some polyphenols which were studied until now. According to the ABTS method, rosmarinic acid, rutin, quercetin, chlorogenic acid, apigenin, chrysin, vitexin, and baicalein have the capability of inhibiting TPO and LOX but with totally diverse potencies. EGCG, curcumin, catechins, biochanin A, apigenin, Pinelliae rhizome, and genistein exerted antioxidative effects via the performance of the antioxidant enzymes which are under the regulation of Nrf2. Their function of inducing Nrf2 activity is in a time- and dose-dependent manner.

## 9. Future Directions

Human studies are often contradictory around the effects of polyphenols against cancer. We believe that it is important to draw boundaries between the uses of polyphenols for cancer prevention versus cancer therapy. This may depend upon various factors, including polyphenols' low bioavailability, their biotransformation, challenging, at colon level and diverse capacity of their circulating metabolites adsorption by specific tissues, leaving open questions for further research. Of note, studies focusing specifically on thyroid cell lines are rare; therefore, further clinical research is required to evaluate the antioxidative activity of polyphenols. Taken together, this review offers insight into the molecular basis of polyphenols activity which may encourage the more widespread application of functional foods as an effective therapy for thyroid cancer. As a future viewpoint for researchers, evaluation of further characterization of the properties of polyphenols related with targeting enzymes may open the way for the utilization of these biomolecules as an adjuvant for the treatment of thyroid cancer. Health-promoting and nutritional properties of herbal compounds are now supported by data coming from nutrigenomics investigations. This offers a powerful approach to discover how nutritional factors affect gene and protein function. The perspective of assessing the genes response to these bioactive compounds in medicine is worthful for further studies. In the future investigations, nutrigenomics and other omics technologies can make this perspective real. The integration of nutrition and genomics may lead to the generation of truly personalized diets for cancer prevention and human health maintenance.

## Figures and Tables

**Table 1:** Antioxidative potential of polyphenols on thyroid models.

Polyphenols	Biomarkers and mechanisms	Models	Ref
Oleuropein	↓Akt ↓ERK ↓H_2_O_2_ ↑ROS	BCPAP and TPC-1	[10]
	Inhibit cell proliferation		
Apigenin	↓Cell division cycle 25C expression ↑ROS Diminished cell viability DNA injury Cell cycle arrest	BCPAP	[60, 61]
Curcumin	↑ROS ↑MMP	K1 PTC	[62]
	↑Intracellular Ca2+ influx		
	Apoptosis Prevention of migration		
Chlorogenic acid Rosmarinic acid Rutin Quercetin	↓Free radicals TPO inhibition	Molecular modeling	[28]
Fisetin Quercetin Morin Kaempferol Myricetin Rutin Naringenin Naringin Catechin Apigenin Sinapinic acid	TPO inhibition	Porcine thyroid glands	[93]
Green tea Cumin Green coffee extract Mustard extract	LOX inhibition	Porcine thyroid glands	[93]
Apigenin	↑p62/Keap1/Nrf2 pathway Nrf2 activation	PTC	[34]
Genistein	↑Nrf2-HO-1/NQO1 pathway Nrf2 activation	FTC-133, NPA, TPC-1, FRO, and ARO	[32, 121, 122, 124]
*Feijoa sellowiana* extract (containing daidzin, genistin, daidzein, genistein, formononetin, biochanin A, and prunetin)	↓Free radicals ↓MDA ↑GSH LPO decreased	Sprague-Dawley male rats	[135]
*Launaea procumbens* extract (containing vitexin, orientin, rutin, hyperoside, catechin, and myricetin)	↑SOD, CAT, and GPx ↓T3 and T4 levels ↓GSH ↓TBARS and H_2_O_2_ LPO decreased	Male albino rats	[136]

**Table 2:** Comparison of observed redox gene expression in rat thyroid cell models with human thyroid tissue and thyroid cancers according to microarray data from oncomine database.

Redox gene		Expression level	Thyroid cancer model
NOX	NOX1 NOX3 NOX5	Minor changes	Papillary and anaplastic thyroid cancers
	NOX2 NOX4	Increased	Papillary and anaplastic thyroid cancers
SOD	SOD1	No change/similar to normal thyroid tissue	Papillary and anaplastic thyroid cancers
	SOD2	Increased	Papillary and anaplastic thyroid cancers
	SOD3	Decreased	Papillary and anaplastic thyroid cancers
CATALASE	CATALASE	Decreased	Anaplastic thyroid cancer
GPX	GPX1 GPX2	Minor increase	Papillary and anaplastic thyroid cancers
	GPX3 GPX5 GPX7	Decreased	Papillary and anaplastic thyroid cancers
	GPX4	No change/similar to normal thyroid tissue	Papillary and anaplastic thyroid cancers
PRDX	PRDX1 PRDX2 PRDX3	Decreased	Papillary and anaplastic thyroid cancers
	PRDX4	Moderately increased	Anaplastic thyroid cancer
	PRDX6	No change/similar to normal thyroid tissue	Papillary and anaplastic thyroid cancers

## Abbreviations

ROS: Reactive oxygen species
GPx1: Glutathione peroxidase
TrxR1: Thioredoxin reductase
*γ*-H2AX: *γ*-H2A histone family member X
53BP1: p53-binding protein 1
DUOX1: Dual oxidase 1
DUOX2: Dual oxidase 2
TPO: Thyroid peroxidase
TC: Thyroid cancer
IFN-*γ*: Interferon gamma
TNF: Tumor necrosis factor
VEGF: Vascular endothelial growth factor
MMPs: Matrix metallopeptidases
COX-2: Cyclooxygenase-2
Mcl-1 protein: Myeloid cell leukemia 1
NF-*κ*B: Nuclear factor kappa-light-chain-enhancer of activated B cells
AGEs: Advanced glycation end products
AOPPs: Advanced oxidation protein products
LOX: Lipoxygenase
MAPK: Mitogen-activated protein kinase
ORAC: Oxygen radical absorbance capacity
FRAP: Ferric reducing antioxidant power
DPPH: 2,2-Diphenyl-1-picrylhydrazy
PCL: Photochemiluminescence
CAA: Cell-based antioxidant assay
OLE: Oleuropein
ERK: Extracellular signal-regulated kinase
Ac-OLE: Acetylated-oleuropein
PTC: Papillary thyroid cancer
AVO: Acidic vesicular organelles
GSH: Glutathione
NOX: NADPH oxidase
TH: Thyroid hormones
ABTS: 2,2′-Azino-bis(3-ethylbenzothiazoline-6-sulfonic acid))
SOD: Superoxide dismutase
CAT: Catalase
GPx: Glutathione peroxidase
GR: Glutathione reductase
Nrf2: Nuclear factor erythroid-related factor-2
ARE: Antioxidant-responsive elements
Keap1: Kelch-like ECH-associated protein 1
AhR: Aryl hydrocarbon receptor
EpRE: Electrophile response element
Tg: Thyroglobulin
TrxR1: Thioredoxin reductase 1
Gpx2: Glutathione peroxidase 2
HO-1: Heme oxygenase-1
STAT1: Signal transducer and activator of transcription 1
LPS: Lipopolysaccharide
NQO-1: NAD(P)H quinone acceptor oxidoreductase 1
GST: Glutathione S-transferase
AMPK: 5′ Adenosine monophosphate-activated protein kinase
PTEN: Phosphatase and tensin homolog
MnSOD: Manganese superoxide dismutase
PUFAs: Polyunsaturated fatty acids.
